# Supplementary material for: Downregulation of the Tumor Suppressor TFF1 Is Required during Induction of Colon Cancer Progression by L1
Source: Cancers (Basel). 2022 Sep 15;14(18):4478. doi: 10.3390/cancers14184478 (PMC9497096; doi:10.3390/cancers14184478)

Table S1: Primers used for qRT-PCR experiments

Gene Name	Forward	Reverse
TFF1	GGACGTCGATGGTATTAGGATA	AGGAGGTAATGGCCACCATGGA
L1	TCGCCCTATGTCCACTACACCT	ATCCACAGGGTTCTTCTCTGGG
GAPDH	GTCTCCTCTGACTTCAACAGCG	ACCACCCTGTTGCTGTAGCCAA

Table S2: List of genes which are either downregulated in L1 overexpressing cells or upregulated in L1+IκB-SR overexpressing cells

Gene Symbol	Gene annotation	Gene description	Fold change	P-value
NFKBIA	NM_020529	Nuclear factor of kappa light polypeptide gene enhancer in B-cells inhibitor, alpha	4.6754	7.28E-27
DRD5	NM_000798	Dopamine receptor D5	3.6852	2.19E-20
C6orf223	BC032706	Chromosome 6 open reading frame 223	2.7512	6.90E-12
FGFBP1	NM_005130	Fibroblast growth factor binding protein 1	2.7218	3.48E-11
TMEM154	NM_152680	Transmembrane protein 154	2.7064	1.17E-12
FGF19	NM_005117	Fibroblast growth factor 19	2.6429	1.69E-11
DDIT4	NM_019058	DNA-damage-inducible transcript 4	2.598	2.32E-10
ZNF117	NM_015852	Zinc finger protein 117	2.5743	4.26E-11
SCD	NM_005063	Stearoyl-CoA desaturase (delta-9-desaturase)	2.4172	1.65E-10
PECR	NM_018441	Peroxisomal trans-2-enoyl-CoA reductase	2.4129	1.83E-09
FAM129A	NM_052966	Family with sequence similarity 129, member A	2.3334	5.57E-06
ERV3	NM_001348050	Endogenous retroviral sequence 3 (includes zinc finger protein H-plk/HPF9)	2.3264	2.35E-08
TRPV6	NM_018646	Transient receptor potential cation channel, subfamily V, member 6	2.3003	1.53E-09

TFF1	NM_003225	Trefoil factor 1	2.2981	6.17E-09
KCNJ5	NM_001354169	Potassium inwardly rectifying channel, subfamily J, member 5	2.2003	6.21E-09
SERPINB8	NM_002640	Serpin peptidase inhibitor, clade B (ovalbumin), member 8	2.2696	8.27E-09
NT5E	NM_002526	5'-nucleotidase, ecto (CD73)	2.232	3.76E-07
CSTA	NM_005213	Cystatin A (stefin A)	2.1885	9.46E-09
CAMK2N1	NM_018584	Calcium/calmodulin-dependent protein kinase II inhibitor 1	2.1883	2.49E-07
LCN2	NM_005564	Lipocalin 2	2.1821	2.63E-07
MCTP1	NM_024717	Multiple C2 domains, transmembrane 1	2.1311	1.31E-07
BHLHB2	NM_003670	Basic helix-loop-helix domain containing, class B, 2	2.0902	9.31E-07
SLFN5	NM_144975	Schlafen family member 5	2.0831	1.42E-06
FLJ16171	AK131247	FLJ16171 protein	2.041	2.75E-07
SMPD3	NM_018667	Sphingomyelin phosphodiesterase 3, neutral membrane (neutral sphingomyelinase II)	2.0505	4.03E-07
LAMP3	NM_014398	Lysosomal-associated membrane protein 3	2.0362	2.11E-06
CHAC1	NM_024111	ChaC, cation transport regulator homolog 1 (E. coli)	2.0208	2.92E-06

Table S3: Percentage of immunohistochemistry results for TFF1-positive phenotypes in 38 cases of CRC

**Phenotype**	**Positive TFF1 signal**
1. Cancer	50% (19/38)
1.1.Mixed Carcinoma	23.7% (9/38)
1.2.Mucinous Carcinoma	26.3% (10/38)
2. Normal	100% (38/38)

Figure S1: Photographs of "scratch wound" closure assays using different clones of LS 174T cells transfected with L1, L1+TFF1, or control (pcDNA3)

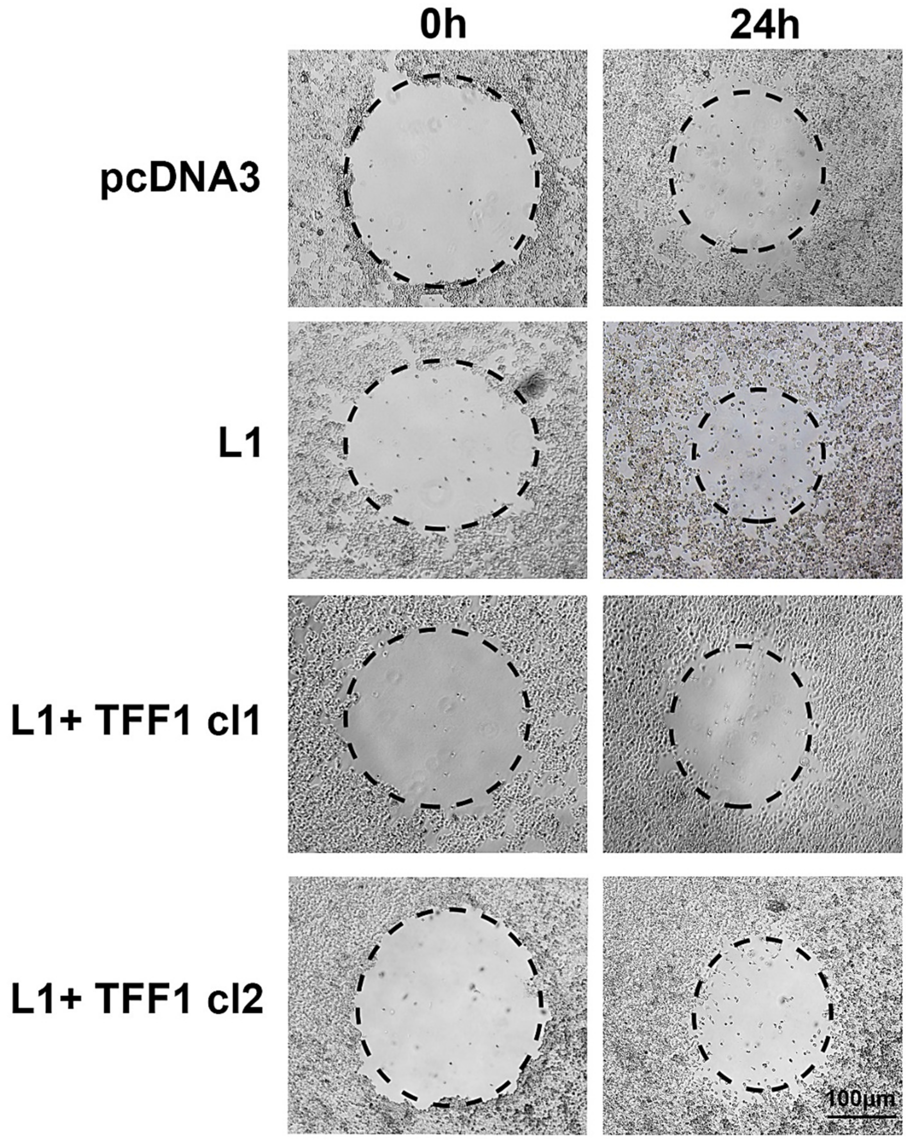

Supplement: Supplementary file 1 [file cancers-14-04478-s001.zip › cancers-1895570-supplementary.pdf]
