# Supplementary material for: Downregulation of the Tumor Suppressor TFF1 Is Required during Induction of Colon Cancer Progression by L1
Source: Cancers (Basel). 2022 Sep 15;14(18):4478. doi: 10.3390/cancers14184478 (PMC9497096; doi:10.3390/cancers14184478)

Figure 1C

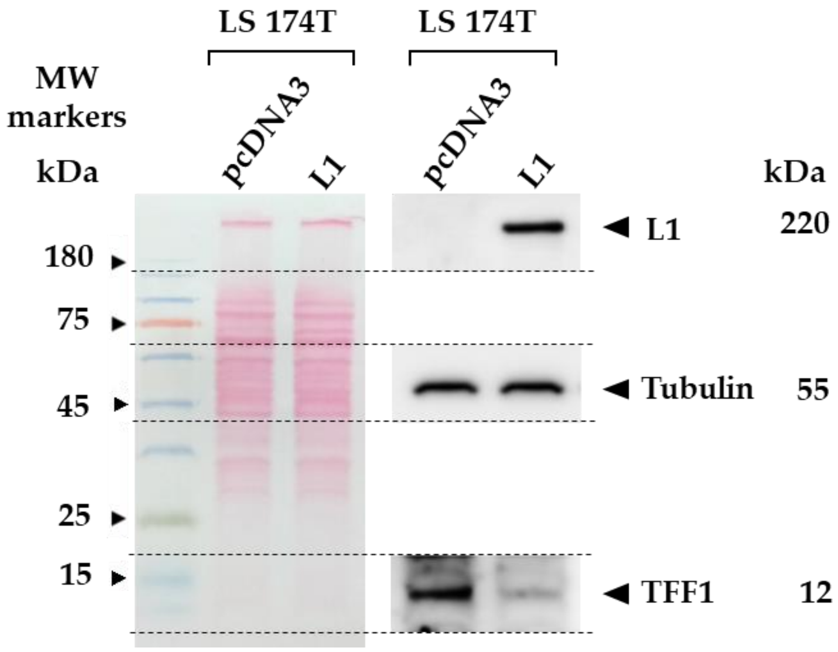

Figure 1D

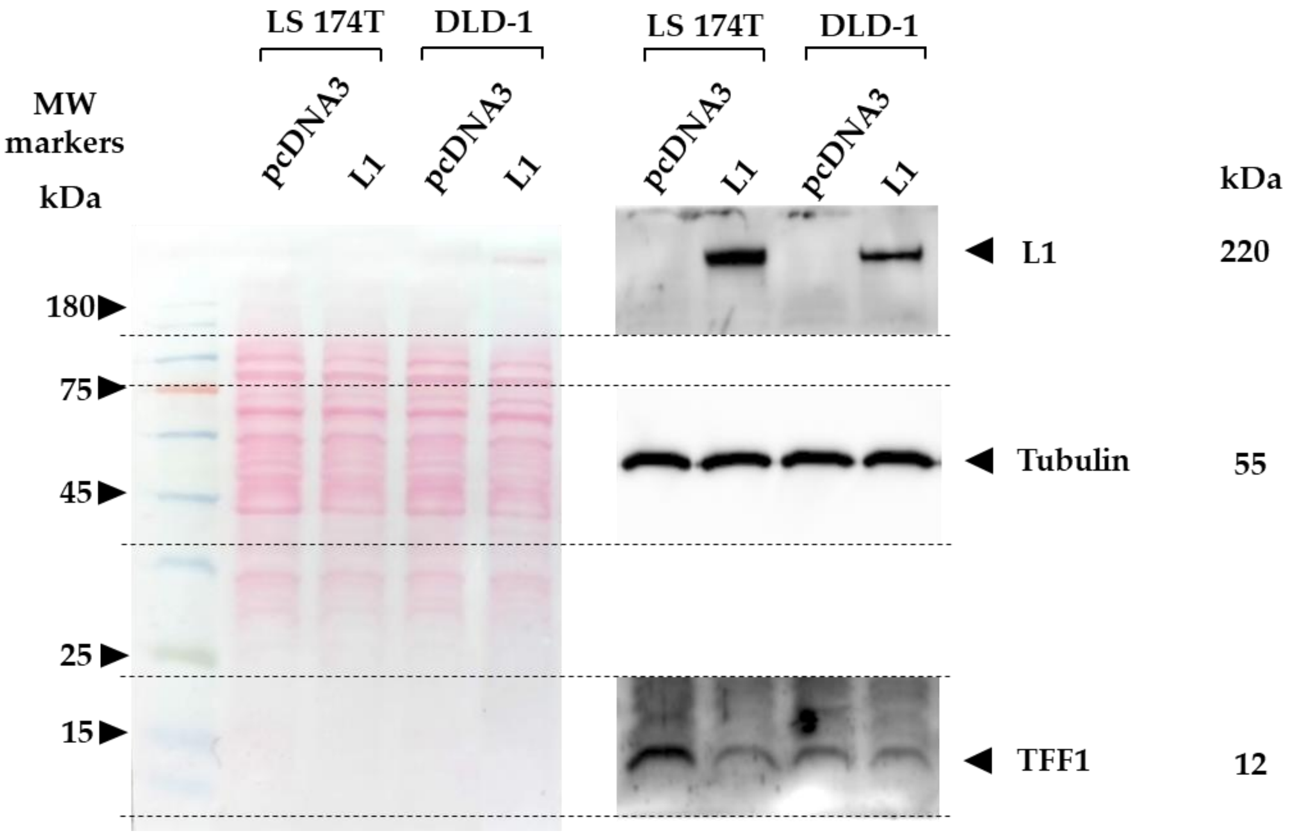

Western blots used to calculate the relative integrated density for TFF1 protein expression in Figure 1E

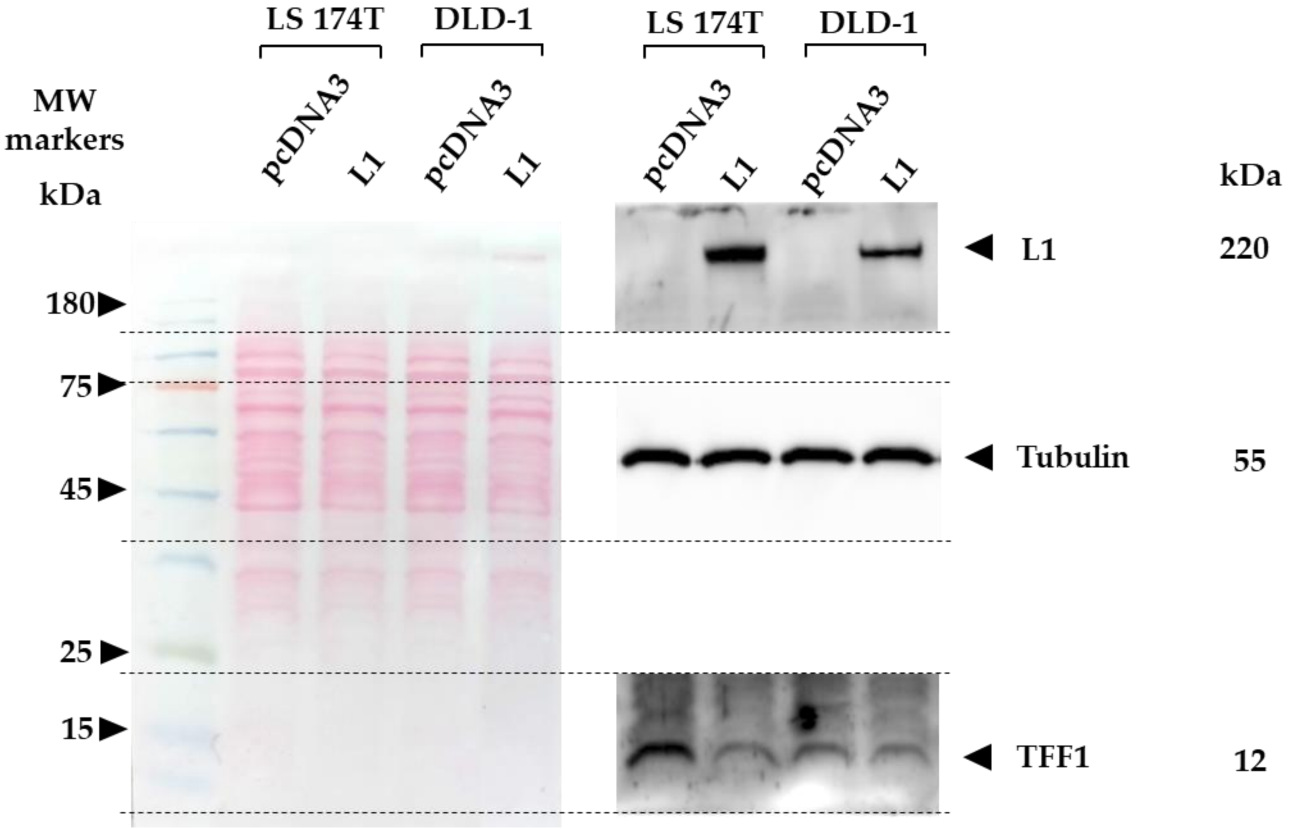

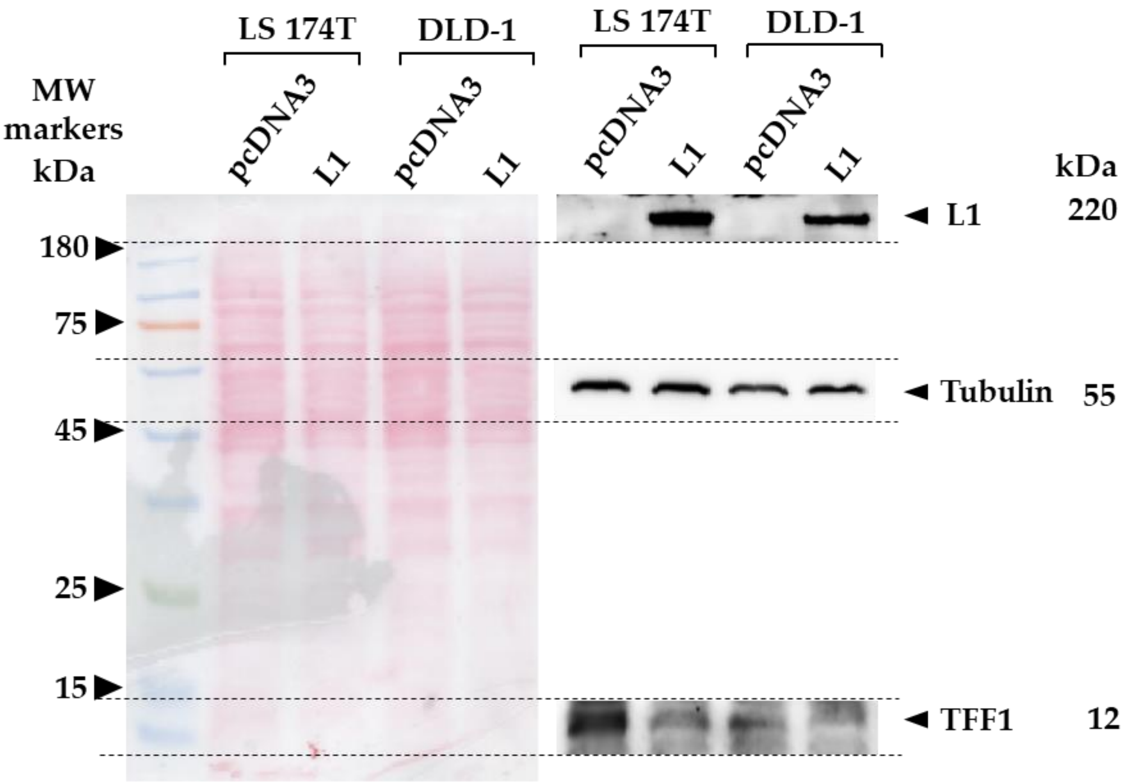

Western blots used to calculate the relative integrated density for TFF1 protein expression in Figure 1E

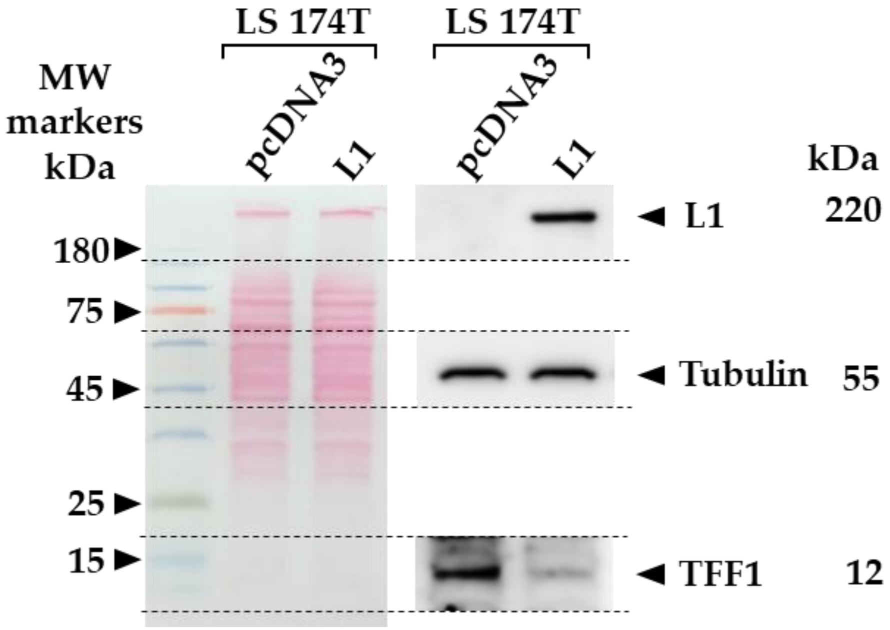

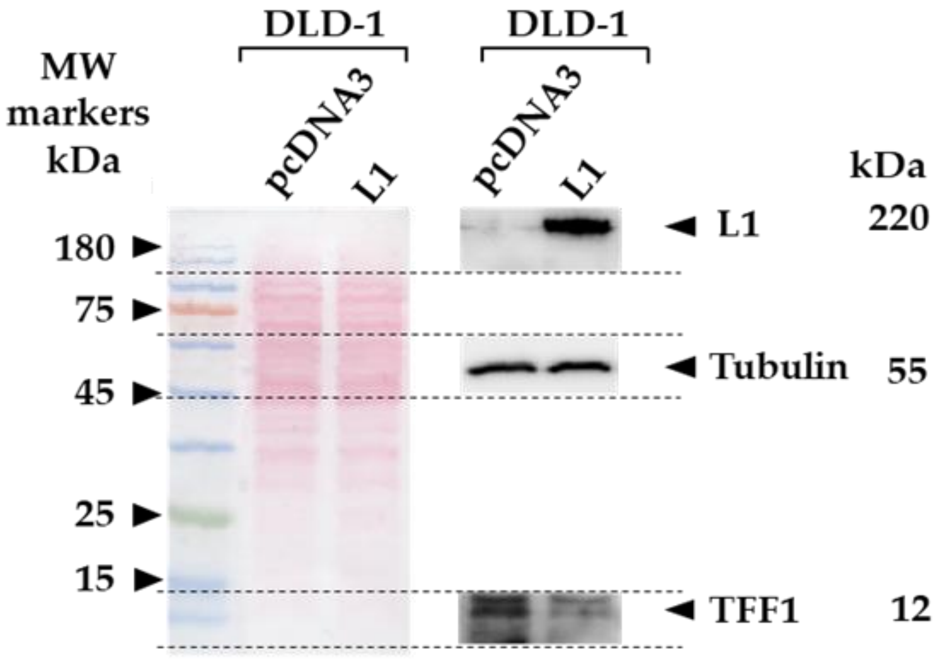

Figure 2A

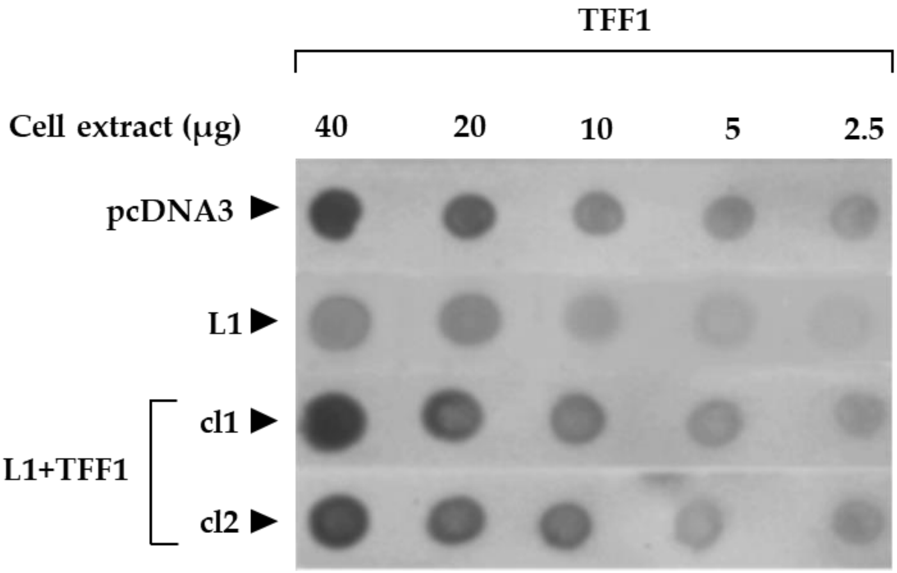

Figure 4A

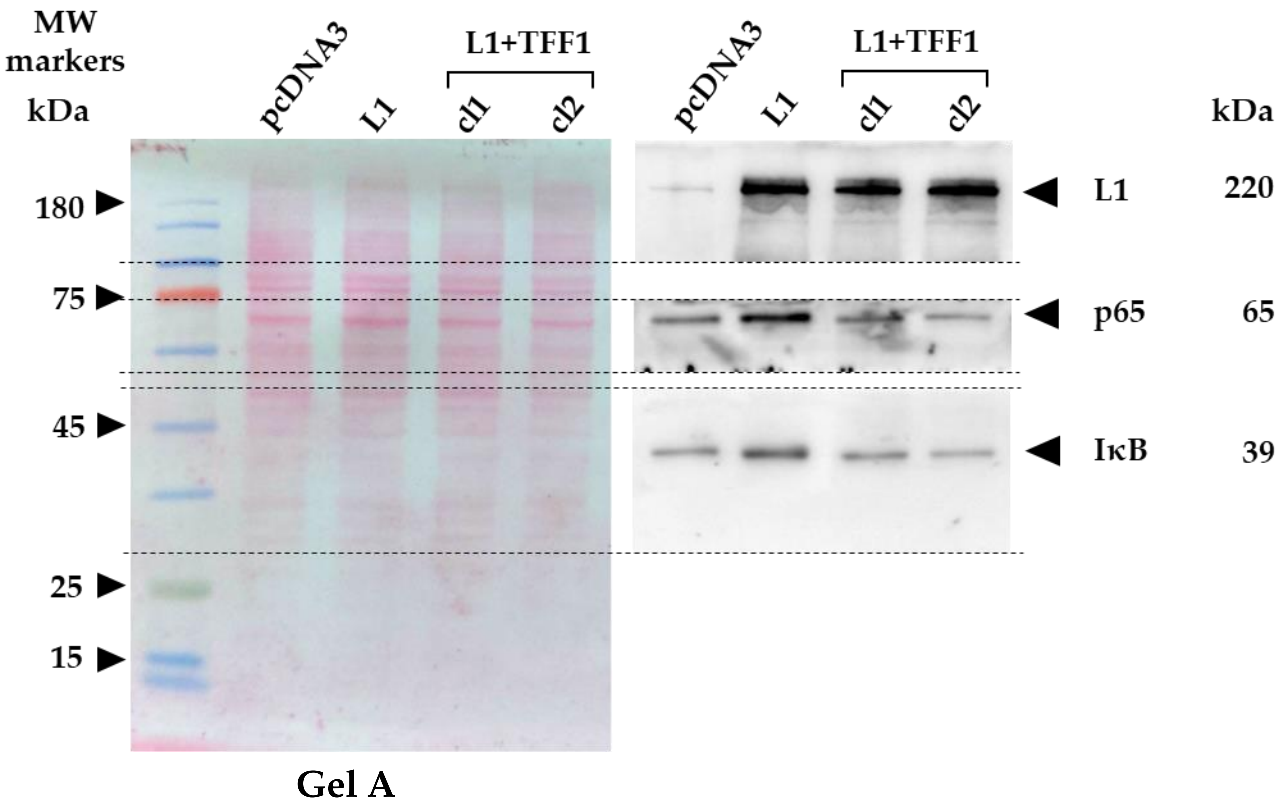

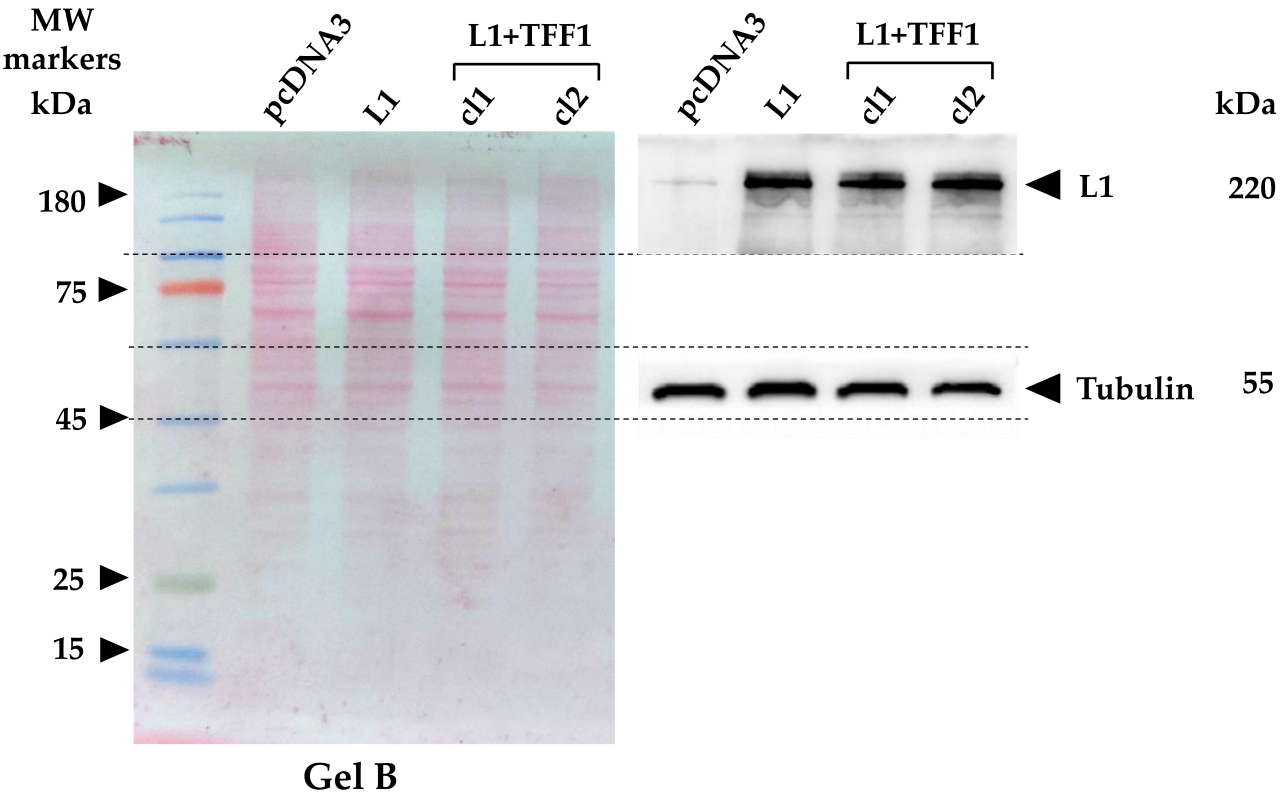

Gel B was re-probed with tubulin

Figure 4C

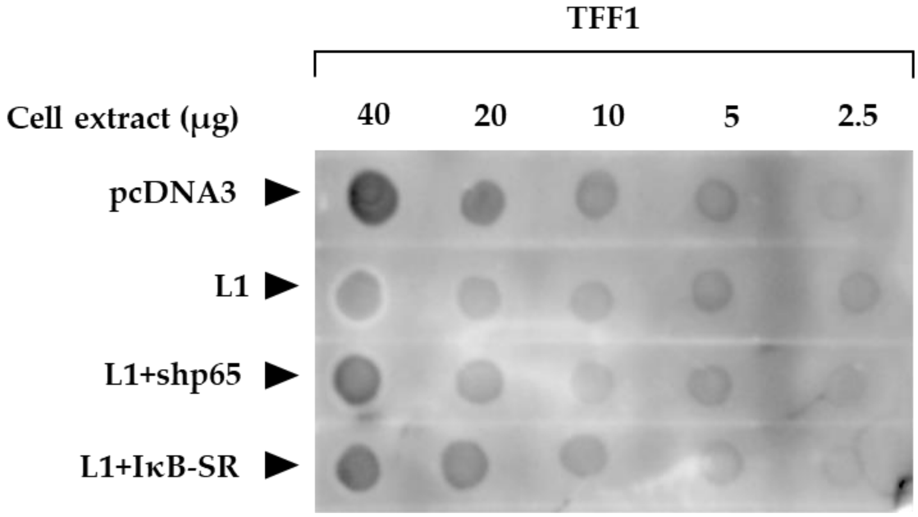

Supplement: Supplementary file 1 [file cancers-14-04478-s001.zip › File S1.pdf]
